# Coffee Drinking

**Published:** 1876-03

**Authors:** 


### COFFEE-DRINKING.

UNTIL chemistry has a better claim than it has to be called one of the exact sciences, we had better give its *dicta* a wide birth as to their practical application in reference to food and drink. Some say that coffee is poisonous because it has strychnine in it. Suppose it is granted that there is strychnine in coffee, and that a single grain of strychnine is so poisonous as to destroy life in a few moments, that does not prove that strychnine in coffee is poisonous, because there may be an element in the coffee which would not only nullify the poisonous quality, but render it absolutely safe, healthful, and nutritious. When a concentrated miasm is mingled with the air a man breathes, he will in a few hours sicken and die; but miasm is of such an intangible aërial nature, that chemistry has no test sufficiently delicate to detect its presence. And if, because chemistry can find no poison there, a man persists in breathing it, he will most certainly suffer the gravest consequences. Several chemists who have had a reputation, and have one still, have certified that they have found no material difference between the milk of farm-house cows and those fed mainly on distillery-swill and confined to filthy apartments. It is true they do not tell us by what process they arrived at these conclusions, but the bare fact of their finding no deleterious substances in the milk of cabined, confined, swill-fed milch cows does not prove that no bad quality existed, but simply that they were not able to detect it, while we all know that just as certainly will infants sicken and die who are fed on swill-milk, as men will sicken and die who breathe a miasmatic atmosphere; so that, in practical cases like these, the masses must be guided in their habits by their observation and their common-sense, and let the vagaries of science and scientific men go out to browse and mature, or, in common phrase, "go to grass."

Thus it is with men of confined views, of limited observation, and still less information, who assert with cool confidence that coffee is poisonous, just as if they knew all of any thing, when really they know all of nothing. The truly learned are cautious in their statements; they fear to deal in other than general expressions of opinion, and leave a bridge of retreat.

They are the worst men in the world to deal in sweeping adjectives — "certain," and "always," and "never," with words like them, are not found in their modest vocabulary; "it appears," "it seems," "it is probable" are frequent phrases in their conversations and writings. As to the bold assertions that coffee is not healthful, is not nutritious, is poisonous, we must appeal to our general observation. People use it daily, and yet live to threescore and ten. For a hundred years past it is more and more used by all who speak the English language, and yet within the last hundred years the average of civilized life is greater by several years; and the Anglo-Saxon race have increased more rapidly than in any other age. To say that they have done this in spite of the increasing use of coffee, and that its ill effects will begin to be felt before a great while, is nothing more than the impudent assertion of a cornered ignoramus.

A single fact sometimes demonstrates a great truth. Within three years, a party bringing the mails from the Rocky Mountains were overtaken by a snow-storm; and in their official report they stated that for two weeks their *entire* subsistence was a few bags of coffee on which they traveled. Had there been no nutriment in the coffee they must have died. To this the anti-coffee men will reply, "We don't know that"— nor do they know any thing else. Meanwhile, if some persons will not use coffee, there will be more left for those who do; and as for ourselves, we ask the liberty of being allowed to eat and drink what we like, and we do most cordially allow that liberty to others, and "no questions asked."

Minute chemical analysis says that the essence of coffee and tea are identical. We believe that both are nutritious and healthful when taken with one restriction as a beverage—never increase it in frequency, strength, or quantity.

---

POINTING WELL.—An article from the JOURNAL OF HEALTH has been going around all creation for nearly a year, commencing thus lucidly: "A person in good health, in the moderate pursuit of business, does not feel like drinking water, even in summer-time, if not very thirsty." We would like to know if any body ever felt like drinking water, summer or winter, if not thirsty. See what nonsense a dot and a letter can make a man talk. It should read thus: "Does not feel like drinking water even in summer, *is* not very thirsty." Make "if" "is," and omit comma after "water."

